# Rarity of Somatic Mutation and Frequency of Normal Sequence Variation Detected in Sporadic Colon Adenocarcinoma Using High-Throughput cDNA Sequencing

**Published:** 2009-11-24

**Authors:** Takatsugu Kan, Bogdan C. Paun, Yuriko Mori, Fumiaki Sato, Zhe Jin, James P. Hamilton, Tetsuo Ito, Yulan Cheng, Stefan David, Alexandru V. Olaru, Jian Yang, Rachana Agarwal, John M. Abraham, Stephen J. Meltzer

**Affiliations:** Gastroenterology Division, Department of Medicine, Johns Hopkins University School of Medicine, 1503 E. Jefferson Street, Rm 106 Baltimore MD 21287

## Abstract

We performed high-throughput cDNA sequencing in colorectal adenocarcinoma and matching normal colorectal epithelium. All six hundred three genes in the UCSC database that were expressed in colon cancers and contained open reading frames of 1000 nucleotides or less were selected for study (total basepairs/bp, 366,686). 304,350 of these 366,686 bp (83.0%) were amplified and sequenced successfully. Seventy-eight sequence variants present in germline (i.e. normal) as well as matching somatic (i.e. tumor) DNA were discovered, yielding a frequency of 1 variant per 3,902 bp. Fifty-one of these sequence variants were homozygous (26 synonymous, 25 non-synonymous), while 27 were heterozygous (11 synonymous, 16 non-synonymous). Cancer tissue contained only one sequence-altered allele of the gene ATP50, which was present heterozygously alongside the wild-type allele in matching normal epithelium. Despite this relatively large number of bp and genes sequenced, no somatic mutations unique to tumor were found. High-throughput cDNA sequencing is a practical approach for detecting novel sequence variations and alterations in human tumors, such as those of the colon.

## Introduction

It is widely believed that somatic as well as germline mutations play important roles in the origin and progression of colorectal cancers (Calvert and Frucht, 2002). Many genes have been investigated for mutation to elucidate mechanisms of colorectal cancer development, with these investigations demonstrating the involvement of mutations in colorectal carcinogenesis and progression. Samuels et al. reported that PIK3CA, a catalytic subunit of the class IA phosphatidylinositol 3-kinases, was somatically mutated in 32% of colorectal cancers, resulting in the attenuation of apoptosis and facilitated tumor invasion(Samuels et al. 2004). A comprehensive study entitled, “The Tyrosine Phosphatome” was accomplished by sequencing all genes involved in tyrosine phosphorylation in a large cancer cohort consisting of 175 colorectal cancer patients(Wang et al. 2004). Most mutational studies, however, have been preoccupied with the prevalence of somatic mutations in a specific single candidate gene in relatively small colorectal cancer patient cohorts. Recently, Sjoblom et al. reported the genome-wide frequencies of somatically mutated genes in human breast and colorectal cancers(Sjoblom et al. 2006). However, the methods these used were extremely expensive, time-consuming, and labor-intensive for a typical laboratory to perform. More practical strategies, amenable to smaller laboratories with more conservative budgets, would be of great value in the continuing quest to answer questions in the fields of tumor genomics and mutatomics. To this end, we present herein a circumscribed, practical mutational study employing high-throughput cDNA sequencing in colon adenocarcinoma, in which we demonstrate the eminent feasibility and results of determining sequence variation efficiently and at low cost.

## Materials and Methods

### Tissue samples

Colorectal cancer and its matching normal colonic mucosa from a patient undergoing surgical resection at the Baltimore VA Hospital after signing informed research consent was used for this study. Clinicopathological data were as follows: 75 year-old male; moderately-differentiated colorectal adenocarcinoma of the ascending colon; tumor size, 2.5 × 1.1 × 0.5 cm; TNM stage (Fifth Edition of the TNM classification of the UICC, 1997), T2N0MX, without any other malignancies. Both colorectal adenocarcinoma and normal colonic epithelium (obtained at the location within the surgically resected specimen furthest from the tumor) were cut into smaller pieces and frozen in liquid nitrogen immediately after removal. A frozen aliquot of each specimen was crushed into pieces and lysed immediately in either TRIZOL reagent (Invitrogen Corp., Carlsbad, CA,) to extract total RNA, or lysis buffer of a DNeasy Tissue kit (QIAGEN Inc., Valencia, CA) to extract DNA, according to these manufacturers’ instructions.

### Cell lines

HeLa S3, HT29, HCT15, HCT116, LoVo, CaCo2, LS174T, LS411N, and DLD1, purchased from the American Type Culture Collection (ATCC), and KYSE30, 70, 110, 150, 220, 410, 770, 850 and OE33, obtained from Dr. Yutaka Shimada at Kyoto University in Japan (Shimada et al. 1992), were enrolled in the current study in order to validate our findings in the ATP50 gene. Culture conditions for each cell line were according to ATCC and the establisher’s recommendations. All cell lines were supplemented with 10% fetal bovine serum plus an appropriate concentration of penicillin and streptomycin.

### Gene selection

To increase our chances of successfully amplifying and sequencing cDNAs, we restricted our study to genes that are known to be expressed in colorectal cancer cells, based on a gene expression database at the University of California, Santa Cruz (UCSC) [http://genome.ucsc.edu/index.html]. From among this gene set, we selected a subset of genes (approximately 600) containing open reading frames (ORFs) 1000 nucleotides or shorter in length. To automate design of the large number of primer sets required, we developed an in-house primer design algorithm based on the publicly available primer design software program, Primer3 (http://frodo.wi.mit.edu/cgi-bin/primer3/primer3_www_slow.cgi). PCR products were designed to range from 300 to 500 bp in length. ORFs of cDNAs longer than 500 bp were divided into 2 or 3 fragments; primers were then designed with adjacent fragments overlapped, in order to completely cover these longer ORFs. Finally, for each heterozygous sequence alteration, genomic DNA primers (available on request) were designed to confirm cDNA sequencing results.

### RT-PCR

Total RNA extracted from colorectal adenocarcinoma and normal colonic epithelium was reverse-transcribed using a SuperScript III First-Strand kit (Invitrogen, Carlsbad, CA), and respective cDNA pools were made. RT-PCR was performed using an AccuPrime Supermix I Kit (Invitrogen). The PCR protocol was as follows: 1 min at 96 °C followed by 35 cycles of 30 sec at 94 °C, 45 sec at 58 °C, and 1 min at 72 °C. Secondary PCR was performed on purified template from the first RT-PCR product, using the same protocol.

### Sequencing

A BigDye Terminator v3.1 Kit (Applied Biosystems, Foster City, CA) was used for the sequencing reaction, and sequence products were read on an SCE 9610 automated 96-capillary sequencer (Spectruby BaseSpectrum v2.10 (SpectruMedix) and analyzed with Mutation Surveyor v2.2 (SoftGenetics LLC, State College, PA). Each time candidate sequence alterations were discovered in cDNA from colorectal cancer tissue, identical procedures were followed in matched normal epitheliam to confirm whether or not they represented somatic alterations. After candidate alterations were confirmed, the entire procedure was repeated separately on a fresh aliquot of cDNA from both the cancer and normal specimens in order to exclude amplification or technical errors due to two-stage PCR. Genomic DNA sequencing was also performed on heterozygous sequence variants to confirm that identical sequence alterations were present in genomic DNA.

### Methylation-specific PCR (MSP)

Because the gene ATP50 was apparently mutated, raising the possibility that it was a tumor suppressor gene, we evaluated this gene for alternative inactivation via promoter hypermethylation. MSP primer sequences of ATP50 for the methylated reaction were: forward (5′-CGAGTGGGAGC-GATTTAGGAC-3′) and reverse (5′-AACGC-CAAAATTACGACACG-3′), which amplify a 94-bp product. β-actin was selected as an internal control gene, using previously published MSP primers (Eads et al. 2001). CpGenome Universal Methylated DNA (Chemicon International, Inc., Temecula, CA) was used as a positive control. The detailed MSP procedure has been previously published (Sato et al. 2002).

### Microsatellite instability (MSI) assay

MSI at each locus was determined by analyses of the length of each PCR-amplified microsatellite. MSI status was confirmed by MSI assays at five consensus loci (BAT25, BAT26, D2S123, D5S346, and D17S250) according to criteria from a National Cancer Institute workshop (Boland et al. 1998). Detailed procedures were as previously described (Mori et al. 2001).

## Results

### Project overview

A total of 603 genes (S-Table 1) were selected based on their length (under 1,000 bp) and their predicted expression in colorectal cancers according to the UCSC database. One thousand thirty-eight primer pairs (available on request) were designed to cover the entire ORFs (total bp, 366,687) of these 603 genes. Sequence data from 862 (83.0%) of these 1,038 primer sets were successfully analyzed, meaning that approximately 304,350 total bp were successfully sequenced (all primer sets for RT-PCR and cDNA sequencing are available on request).

### Sequence variants

**Figure 1.**
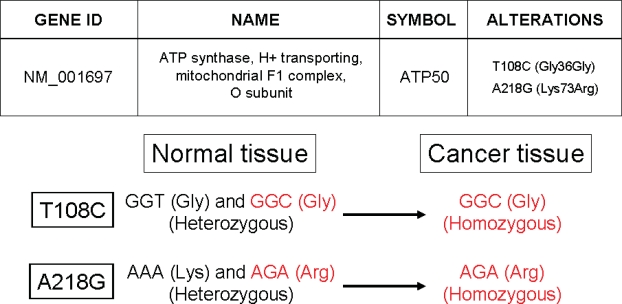
**cDNA sequencing of ATP50.** Two different alteration sites were detected. At the 108th nucleotide, colorectal cancer tissue had only a mutant cytosine nucleotide, while normal colon contained both a thymine (wild) and a cytosine (mutant). Both codons GGT and GGC encoded glycine (synonymous alteration). At the 218th mucleotide, colorectal cancer tissue had only a mutant guanine nucleotide, while normal tissue contained both an adenine (wild) and a guanine (mutant). AAA encoded lysine and AGA encoded arginine (non-synonymous alteration). *Gly*, glycine; *Arg*, arginine.

Seventy-eight sequence variants within 50 genes were found among the 603 genes studied (Table 1) (S-Table 2 for detailed information). Thus, the frequency of sequence variants was 1 per 3,902 bp (78 total variants/304,350 total bp). Of these 78 sequence alterations, 51 were homozygous (26 synonymous, 25 non-synonymous) and 27 were heterozygous (11 synonymous, 16 non-synonymous). All sequence alterations were detected in both colorectal cancer tissue and matched normal colonic epithelium, with the exception of an alteration in the gene ATP50 (NM_001697), which manifested a unique expression mechanism (Fig. 1). Forty-four sequence alterations had been previously reported, but 34 sequence alterations were completely novel, having never been reported in the SNP database at The National Center for Biotechnology Information (NCBI).

### Tumor-specific regulation of gene expression

**Figure 2.**
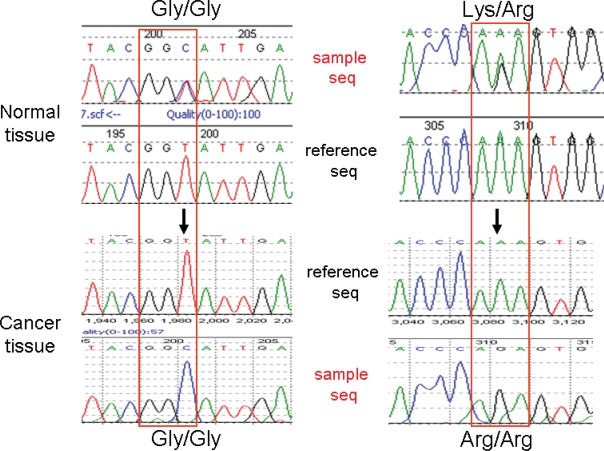
**Representative result of cDNA sequencing of ATP50 in normal and cancer tissues.** The *red box* in the *left panel* shows the 36th codon, while the *red box* in the *right panel* shows the 73rd codon. All alterations were confirmed by both forward and reverse sequencing.

**Figure 3.**
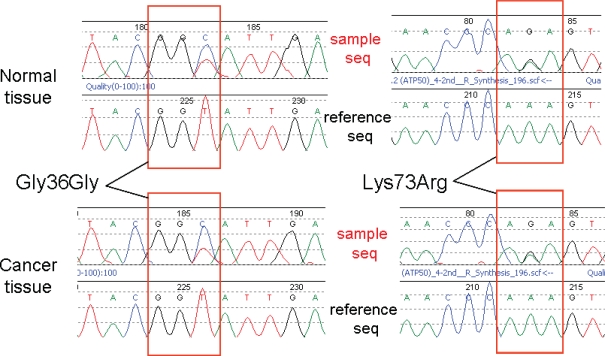
**Genomic DNA sequencing of ATP50 in normal and cancer tissues.** *Upper panels,* forward sequencing; *lower panels,* reverse sequencing. The *red boxes* show the 36th and 73rd codons. Both normal and colorectal cancer tissues contain identical heterozygosities at the 108th and 218th nucleotides.

Tumor-specific regulation of gene expression was found for NM_001697 (ATP50, Homo sapiens ATP synthase, H+ transporting, mitochondrial F1 complex, O subunit). The sequence alterations T108C (GGT to GGC, homozygous, Gly36Gly) and A218G (AAA to AAG, homozygous, Lys73Arg) were observed only in cancer-derived cDNA, while the alterations T108TC (CGT and GGC, heterozygous, 36Gly) and A218AG (AAA and AAG, heterozygous, 73Lys and 73 Arg) were observed in cDNA from normal epithelium. Surprisingly, both T108TC (CGT and GGC, heterozygous, 36Gly) and A218AG (AAA and AAG, heterozygous, 73Lys and 73 Arg), which were identical to the two alterations observed in normal cDNA, were observed in genomic DNA from both cancer and normal tissue (Figures 2, 3). This result implied that the cancer exhibited monoallelic expression from the variant allele of ATP50, while the normal epithelium manifested biallelic heterozygous expression, i.e. from both the reported normal allele and our discovered variant mutant allele simultaneously.

### MSP

One possible mechanism for monoallelic expression observed for ATP50 was DNA methylation of its promoter region. MSP showed, however, that there was no methylation of the ATP50 promoter in colorectal cancer (S-Fig. 1).

### Somatic mutations

There were no somatic mutations found among the 603 genes studied or within the p53 gene.

### MSI status

MSI assays showed that there was no microsatellite instability in genomic DNA (S-Fig. 2).

## Discussion

In the current study, we assumed that if a mutant protein was involved in carcinogenesis or tumor progression, this mutant would be expressed and therefore detectable in tumor mRNA. i.e. we assumed that somatic mutations involved in carcinogenesis or tumor progression would be detectable by direct cDNA sequencing. By using this strategy, we avoided the need for sequencing each exon of genomic DNA, reasoning that genes which are never expressed in normal or malignant colon probably do not participate in colorectal carcinogenesis. We discovered 78 sequence variants (44 of which had been previously reported as single-nucleotide polymorphisms, but 34 of which had never been reported) among the 603 genes (304,350 bp of ORFs) studied.

Recently, Sjoblom T. et al. performed genome-wide sequencing in breast and colorectal cancers, revealing that an average of 52 mutations occurred in each colorectal cancer(Sjoblom et al. 2006). According to the article by Sjoblom et al. the somatic mutation frequency in colon cancers was 3.2 somatic mutations/Mb, on average (Table 1 of their paper). Therefore, the probability of our finding zero somatic mutations among the 603 genes (304,350 bp) that we studied was 37.76% (please see formula below), suggesting that our findings were statistically quite consistent with Sjoblom’s results:

$$\begin{array}{l} \text{Probability}={\left(1-\frac{3.2}{1,\;000,\;000}\right)}^{304,350}\\ \;\;\;\;\;\;\;\;\;\;\;\;\;\;\;\;\;\;\;\;\;\;\;\;\;\;\;\;\;\;\;\;\;\;\;\;\;\;\;\;\;\;\;=0.377599353 \end{array}$$

The Sjoblom team also defined “CAN-genes” (candidate cancer genes) as those that were frequently mutated in colorectal cancers, and found that 69 genes could be included in this category. Although the CAN-genes KRAS, GNAS and TP53 were studied by us, no somatic mutations were found in these genes. Furthermore, in addition to the genes mentioned above, NRAS, HRAS, p16, and p27 were included in the current study, but these genes also contained no somatic mutations. Finally, results of MSI assays revealed MS-stability (MSS), implying an absence of mutations in the major DNA mismatch repair genes (although these genes were not studied due to their long ORFs). It is possible that other molecular pathogenetic pathways were involved in this colorectal tumorigenesis, such as those containing APC, MCC, DCC, or the TGF-β cascade: these genes were also not examined in the current study due to ORF length.

Approximately 24,000,000 bp among the entire genomic DNA sequence are reported as ORFs in the UCSC database. The average density of each SNP is once per 1.9 kilobases (i.e. 1,419,190 SNPs/2.7 gigabases of human genome sequence)(Sachidanandam et al. 2001). We sequenced 304,350 bp of ORFs (*viz.,* 1.26% of the total ORFs in the UCSC database: 304,350 bp/24,000,000 bp) and discovered 78 sequence variants, yielding a frequency of 1 alteration per 3,902 bp (78/304,350 bp). Our observed sequence variant distribution may provide a basis with which to estimate the number of SNPs in a single individual with colon cancer. That is, the SNPs reported above are one possible subset of the entire database; there is no guarantee that a given individual will always harbor all SNPs in the database.

The human ATP50 gene (X83218, NM_001697), encoding a 213-amino acid ATP synthase OSCP subunit, is a key structural component of the stalk of the mitochondrial respiratory chain F_1_F_0_-ATP synthase, which is a vital element in the cellular pathway of energy conversion (Senior, 1988). Although a mutant strain of yeast in which the delta subunit of F_1_F_0_-ATP synthase had been inactivated by insertional mutagenesis showed little or no ATPase activity(Giraud and Velours, 1994), and dysfunction of ATP synthase can cause a variety of degenerative diseases(Wallace, 1994), there have been no previous reports detailing a relationship between ATP synthase and tumorigenesis. We found restricted monoallelic (i.e. monoallelically silenced) expression of an altered allele from ATP50 in our colon cancer tissue, which would be expected to exert the same effect as would a somatic mutation of this gene. Genomic DNA sequencing of ATP50 revealed that this monoallelic expression was not due to LOH. We therefore studied the methylation status of the CpG island in the promoter region of ATP50 by MSP, but we found no methylation of this region. Other epigenetic mechanisms, such as histone deacetylation, might have contributed to monoallelic expression of ATP50. There was no monoallelic expression of ATP50 in 20 cancer cell lines that we examined. Although monoallelic expression of this altered ATP50 allele may be involved in a subset of colorectal cancers, further study is required to clarify the potential functional role of this gene in carcinogenesis.

This study poses several advantages as well as limitations. Firstly, it has been reported that some synonymous mutations may influence the stability of mRNA(Duan and Antezana, 2003; Chamary and Hurst, 2005) because they affect the thermodynamic stability of mRNA secondary structures (Fitch, 1974; Klambt, 1975). Nonsense-mediated mRNA decay (NMD) is also known as a surveillance pathway that rapidly degrades mRNAs containing premature termination codons(Culbertson and Leeds, 2003; Amrani et al. 2006). These mechanisms may cause instability of mRNA, accelerate the degradation of mRNA, and consequently result in difficulty in detecting sequence alterations by cDNA sequencing. Since we used cDNA as our starting material for sequencing, we may have ignored some key genes because of RNA degradation. Nevertheless, many sequence variants were detected reasonably well in the current study, suggesting that degradation of mRNA occurred rarely, if at all, as a consequence of sequence alterations. Instead, we considered it more important to increase our chances of finding sequence alterations by using cDNA rather than genomic DNA because of the lower cost, time, and labor involved in sequencing cDNA, as well as the increased relevance of only studying genes that are expressed in the colon.

Secondly, it is conceivable that we lost some gene sequence information due to extremely low expression levels. Therefore, we employed two-stage PCR to increase our chances of successful sequencing, thereby achieving a relatively high success rate of 862/1,038 reactions, or 83.0%. Possibly, this result still may have included genes that were not expressed in our particular colorectal cancer, even though we used the UCSC database to select genes that were purportedly expressed in colorectal cancers. Our sequencing success rate appears favorable when compared to genomic DNA sequencing, where 92% of genes were successfully analyzed (Wang et al. 2004). The total number of exons sequenced in our study was 2107, implying that at least 2107 primer pairs would have been necessary to conduct this study had it been attempted by genomic DNA sequencing; in contrast, we accomplished this task using only 1038 primer sets for cDNA sequencing. This contrast demonstrates that our method is useful to explore mutations because it is not only more cost-effective, but also less demanding in time and labor.

## Supplement Material

S-Figure 1. Result of MSP for ATP50. (A) Bisulfited DNA from cancer tissue, normal tissue, and HT29 were used. The MSPs for ATP50 were triplicated. The MSP product for ATP50 was 94 bp and beta actin was 133bp. (B) Universal methylated DNA was used for the positive control in various amounts. M.W., molecular weight.

S-Figure 2. A representative result of MSI typing. Both cancer and normal tissue showed microsatellite stable in the marker of D5S346 and D17S250. The similar results were obtained in the marker of D2S123, BAT25, and BAT26.

**S-Table-1.** Gene list

**GenBank Accession No.**	**Gene Symbol**	**GenBank Accession No.**	**Gene Symbol**	**GenBank Accession No.**	**Gene Symbol**
**NM_000100.2**	CSTB	**NM_198057.1**	TSC22D3	**NM_002811.3**	PSMD7
**NM_000999.2**	RPL38	**NM_022652.2**	DUSP6	**NM_002812.3**	PSMD8
**NM_001827.1**	CKS2	**NM_021121.2**	EEF1B2	**NM_002813.4**	PSMD9
**NM_001863.3**	COX6B1	**NM_001412.2**	EIF1AX	**NM_170750.1**	PSMD10
**NM_001866.2**	COX7B	**NM_004094.3**	EIF2S1	**NM_004577.3**	PSPH
**NM_001867.2**	COX7C	**NM_001423.1**	EMP1	**NM_175847.1**	PTBP1
**NM_002489.2**	NDUFA4	**NM_001424.3**	EMP2	**NM_198974.1**	PTK9
**NM_002491.1**	NDUFB3	**NM_001425.1**	EMP3	**NM_002823.2**	PTMA
**NM_002966.1**	S100A10	**NM_207168.1**	ENSA	**NM_002824.4**	PTMS
**NM_003009.2**	SEPW1	**NM_001005915.1**	ERBB3	**NM_133377.1**	RAD1
**NM_003063.1**	SLN	**NM_001983.2**	ERCC1	**NM_153824.1**	PYCR1
**NM_003095.1**	SNRPF	**NM_001984.1**	ESD	**NM_000320.1**	QDPR
**NM_003133.1**	SRP9	**NM_001439.1**	EXTL2	**NM_004161.3**	RAB1A
**NM_003498.3**	SNN	**NM_001997.2**	FAU	**NM_004162.3**	RAB5A
**NM_003746.1**	DYNLL1	**NM_005247.2**	FGF3	**NM_002868.2**	RAB5B
**NM_003860.2**	BANF1	**NM_002007.1**	FGF4	**NM_198896.1**	RAB6A
**NM_003945.3**	ATP6V0E	**NM_002010.1**	FGF9	**NM_002870.2**	RAB13
**NM_004045.2**	ATOX1	**NM_023108.1**	FGFR1	**NM_183235.1**	RAB27A
**NM_004485.2**	GNG4	**NM_001449.3**	FHL1	**NM_201434.1**	RAB5C
**NM_004541.2**	NDUFA1	**NM_054014.1**	FKBP1A	**NM_198829.1**	RAC1
**NM_004772.1**	C5orf13	**NM_002013.2**	FKBP3	**NM_002872.3**	RAC2
**NM_005274.1**	GNG5	**NM_016725.1**	FOLR1	**NM_133630.1**	RAD51L3
**NM_005517.2**	HMGN2	**NM_004477.1**	FRG1	**NM_002881.2**	RALB
**NM_005694.1**	COX17	**NM_000146.2**	FTL	**NM_006325.2**	RAN
**NM_005770.3**	SERF2	**NM_198903.1**	GABRG2	**NM_002884.1**	RAP1A
**NM_005887.1**	DLEU1	**NM_000166.2**	GJB1	**NM_015646.3**	RAP1B
**NM_005949.1**	MT1F	**NM_024009.2**	GJB3	**NM_032626.5**	RBBP6
**NM_005954.2**	MT3	**NM_002061.2**	GCLM	**NM_181558.1**	RFC3
**NM_005978.3**	S100A2	**NM_006708.1**	GLO1	**NM_181578.1**	RFC5
**NM_006156.1**	NEDD8	**NM_002066.1**	GML	**NM_134427.1**	RGS3
**NM_006274.2**	CCL19	**NM_016592.1**	GNAS	**NM_005614.2**	RHEB
**NM_006304.1**	SHFM1	**NM_005301.2**	GPR35	**NM_000326.3**	RLBP1
**NM_006353.2**	HMGN4	**NM_002083.2**	GPX2	**NM_002938.2**	RNF4
**NM_006698.2**	BLCAP	**NM_002084.2**	GPX3	**NM_183045.1**	RNF6
**NM_006829.2**	C10orf116	**NM_203506.1**	GRB2	**NM_002946.3**	RPA2
**NM_007233.1**	TP53AP1	**NM_001512.2**	GSTA4	**NM_002947.3**	RPA3
**NM_007281.1**	SCRG1	**NM_147149.1**	GSTM4	**NM_033301.1**	RPL8
**NM_012456.1**	TIMM10	**NM_145871.1**	GSTZ1	**NM_033251.1**	RPL13
**NM_012458.2**	TIMM13	**NM_004492.1**	GTF2A2	**NM_000985.2**	RPL17
**NM_012460.2**	TIMM9	**NM_002095.3**	GTF2E2	**NM_000984.3**	RPL23A
**NM_013332.1**	HIG2	**NM_000858.3**	GUK1	**NM_000992.2**	RPL29
**NM_013343.1**	LOH3CR2A	**NM_005318.2**	H1F0	**NM_001001.3**	RPL36AL
**NM_014041.1**	SPCS1	**NM_002106.3**	H2AFZ	**NM_021029.3**	RPL36A
**NM_014051.2**	TMEM14A	**NM_005324.3**	H3F3B	**NM_001002.3**	RPLP0
**NM_014221.1**	MTCP1	**NM_005326.3**	HAGH	**NM_002949.2**	MRPL12
**NM_014356.2**	C6orf123	**NM_005327.1**	HADHSC	**NM_001007.3**	RPS4X
**NM_014445.2**	SERP1	**NM_005330.3**	HBE1	**NM_001015.3**	RPS11
**NM_014624.3**	S100A6	**NM_004494.1**	HDGF	**NM_001019.3**	RPS15A
**NM_014792.2**	KIAA0125	**NM_139011.1**	HFE	**NM_001020.3**	RPS16
**NM_016096.2**	ZNF706	**NM_005340.3**	HINT1	**NM_001022.3**	RPS19
**NM_016305.1**	SS18L2	**NM_002118.3**	HLA-DMB	**NM_001023.2**	RPS20
**NM_016565.2**	CHCHD8	**NM_002128.3**	HMGB1	**NM_001025.3**	RPS23
**NM_020142.3**	LOC56901	**NM_002129.2**	HMGB2	**NM_002960.1**	S100A3
**NM_020179.1**	FN5	**NM_004965.6**	HMGN1	**NM_005620.1**	S100A11
**NM_020181.1**	C14orf162	**NM_002131.2**	HMGA1	**NM_000664.3**	ACACA
**NM_020248.1**	CTNNBIP1	**NM_173158.1**	NR4A1	**NM_198970.1**	AES
**NM_020408.3**	C6orf149	**NM_002136.1**	HNRPA1	**NM_001636.1**	SLC25A6
**NM_021104.1**	RPL41	**NM_031314.1**	HNRPC	**NM_001001787.1**	ATP1B1
**NM_021127.1**	PMAIP1	**NM_002138.3**	HNRPD	**NM_001687.4**	ATP5D
**NM_021177.3**	LSM2	**NM_021644.2**	HNRPH3	**NM_001002256.1**	ATP5G3
**NM_023937.2**	MRPL34	**NM_006896.2**	HOXA7	**NM_004047.2**	ATP6V0B
**NM_031286.2**	SH3BGRL3	**NM_153715.1**	HOXA10	**NM_198589.1**	BSG
**NM_031287.2**	SF3B5	**NM_156037.1**	HOXB6	**NM_004927.2**	MRPL49
**NM_032412.2**	ORF1-FL49	**NM_004502.2**	HOXB7	**NM_006136.2**	CAPZA2
**NM_032574.1**	LOC84661	**NM_024016.2**	HOXB8	**NM_022845.2**	CBFB
**NM_032747.1**	USMG5	**NM_014620.2**	HOXC4	**NM_001760.2**	CCND3
**NM_052871.2**	MGC4677	**NM_153693.1**	HOXC6	**NM_171827.1**	p32/CD8A
**NM_052971.1**	LEAP-2	**NM_006897.1**	HOXC9	**NM_001773.1**	CD34
**NM_080677.1**	DYNLL2	**NM_014212.2**	HOXC11	**NM_000611.4**	CD59
**NM_138448.2**	ACYP2	**NM_024501.1**	HOXD1	**NM_001780.3**	CD63
**NM_139286.3**	CDC26	**NM_134421.1**	HPCAL1	**NM_004359.1**	CDC34
**NM_194327.1**	GALIG	**NM_182638.1**	HPS1	**NM_058197.2**	p16/CDKN2A
**NM_198835.1**	ACACA	**NM_005524.2**	HES1	**NM_001280.1**	CIRBP
**NM_020115.3**	ACRV1	**NM_198431.1**	HSPA4	**NM_001833.1**	CLTA
**NM_001124.1**	ADM	**NM_001540.2**	HSPB1	**NM_022645.2**	CSH2
**NM_000674.1**	ADORA1	**NM_005528.1**	DNAJC4	**NM_012140.3**	SLC25A10
**NM_000676.2**	ADORA2B	**NM_181353.1**	ID1	**NM_148979.1**	CTSH
**NM_001630.1**	ANXA8	**NM_002166.4**	ID2	**NM_000396.2**	CTSK
**NM_001154.2**	ANXA5	**NM_174856.1**	IDH3B	**NM_001336.2**	CTSZ
**NM_080649.1**	APEX1	**NM_004508.2**	IDI1	**NM_001915.2**	CYB561
**NM_000041.2**	APOE	**NM_005533.2**	IFI35	**NM_004418.2**	DUSP2
**NM_152876.1**	FAS	**NM_021068.1**	IFNA4	**NM_004427.2**	PHC2
**NM_000486.3**	AQP2	**NM_000612.2**	IGF2	**NM_001970.3**	EIF5A
**NM_053286.1**	AQP6	**NM_001552.1**	IGFBP4	**NM_001419.2**	ELAVL1
**NM_001659.1**	ARF3	**NM_000576.2**	IL1B	**NM_198194.1**	STOM
**NM_001660.2**	ARF4	**NM_172200.1**	IL15RA	**NM_202001.1**	ERCC1
**NM_001663.2**	ARF6	**NM_005536.2**	IMPA1	**NM_023110.1**	FGFR1
**NM_001664.2**	RHOA	**NM_014214.1**	IMPA2	**NM_201557.1**	FHL2
**NM_004040.2**	RHOB	**NM_198219.1**	ING1	**NM_004468.3**	FHL3
**NM_175744.3**	RHOC	**NM_198337.1**	INSIG1	**NM_057092.1**	FKBP2
**NM_005168.2**	RND3	**NM_002198.1**	IRF1	**NM_016730.1**	FOLR1
**NM_001665.2**	rho G	**NM_004030.1**	IRF7	**NM_004477.1**	FRG1
**NM_004309.3**	ARHGDIA	**NM_181493.1**	ITPA	**NM_002032.1**	FTH1
**NM_001177.3**	ARL1	**NM_002228.3**	JUN	**NM_002035.1**	FVT1
**NM_004311.2**	ARL3	**NM_002231.2**	CD82	**NM_001487.1**	BLOC1S1
**NM_004314.1**	ART1	**NM_004137.2**	KCNMB1	**NM_004483.3**	GCSH
**NM_032468.2**	ASPH	**NM_033360.2**	KRAS	**NM_004124.2**	GMFB
**NM_005171.2**	ATF1	**NM_002295.2**	RPSA	**NM_000581.2**	GPX1
**NM_004024.2**	ATF3	**NM_005563.3**	STMN1	**NM_002085.1**	GPX4
**NM_001677.3**	ATP1B1	**NM_005564.2**	LCN2	**NM_147148.1**	GSTM4
**NM_001679.2**	ATP1B3	**NM_005566.1**	LDHA	**NM_002095.3**	GTF2E2
**NM_001001977.1**	ATP5E	**NM_201544.1**	LGALS8	**NM_002107.3**	H3F3A
**NM_001002015.1**	ATP5F1	**NM_004987.3**	LIMS1	**NM_005342.1**	HMGB3
**NM_005175.2**	ATP5G1	**NM_005574.2**	LMO2	**NM_002133.1**	HMOX1
**NM_001002258.1**	ATP5G3	**NM_002346.1**	LY6E	**NM_002134.2**	HMOX2
**NM_001003701.1**	ATP5J	**NM_002353.1**	TACSTD2	**NM_156036.1**	HOXB6
**NM_001694.2**	ATP6V0C	**NM_014220.1**	TM4SF1	**NM_024017.3**	HOXB9
**NM_001697.2**	ATP5O	**NM_002354.1**	TACSTD1	**NM_000194.1**	HPRT1
**NM_004322.2**	BAD	**NM_030885.2**	MAP4	**NM_005343.2**	HRAS
**NM_053056.1**	CCND1	**NM_203378.1**	MB	**NM_174856.1**	IDH3B
**NM_138578.1**	BCL2L1	**NM_002386.2**	MC1R	**NM_000628.3**	IL10RB
**NM_004050.2**	BCL2L2	**NM_182763.1**	MCL1	**NM_181431.1**	FOXK2
**NM_000713.1**	BLVRB	**NM_012328.1**	DNAJB9	**NM_181468.1**	ITGB4BP
**NM_005180.5**	PCGF4	**NM_005370.4**	RAB8A	**NM_201543.1**	LGALS8
**NM_004331.2**	BNIP3L	**NM_177524.1**	MEST	**NM_002359.2**	MAFG
**NM_032515.3**	BOK	**NM_005371.3**	METTL1	**NM_004528.2**	MGST3
**NM_004332.1**	BPHL	**NM_017459.1**	MFAP2	**NM_022792.2**	MMP19
**NM_007306.1**	BRCA1	**NM_145791.1**	MGST1	**NM_002448.1**	MSX1
**NM_198590.1**	BSG	**NM_002413.3**	MGST2	**NM_005962.3**	MXI1
**NM_001207.3**	BTF3	**NM_002414.3**	CD99	**NM_079424.1**	MYL6
**NM_001731.1**	BTG1	**NM_002415.1**	MIF	**NM_032104.1**	PPP1R12B
**NM_007311.2**	BZRP	**NM_022791.2**	MMP19	**NM_004547.4**	NDUFB4
**NM_172369.1**	C1QG	**NM_002434.1**	MPG	**NM_182739.1**	NDUFB6
**NM_001217.2**	CA11	**NM_021126.3**	MPST	**NM_005005.1**	NDUFB9
**NM_000387.3**	SLC25A20	**NM_012331.2**	MSRA	**NM_020529.1**	NFKBIA
**NM_006888.2**	CALM1	**NM_002451.3**	MTAP	**NM_002520.4**	NPM1
**NM_005184.1**	CALM3	**NM_015675.1**	GADD45B	**NM_002607.2**	PDGFA
**NM_005185.2**	CALML3	**NM_001002841.1**	(MYL4	**NM_005022.2**	PFN1
**NM_001745.2**	CAMLG	**NM_079423.1**	MYL6	**NM_000942.4**	PPIB
**NM_001003962.1**	CAPNS1	**NM_002478.3**	MYOD1	**NM_206873.1**	PPP1CA
**NM_004346.2**	CASP3	**NM_032103.1**	PPP1R12B	**NM_183079.1**	PRNP
**NM_001755.2**	CBFB	**NM_005594.2**	NACA	**NM_002765.2**	PRPS2
**NM_004059.3**	CCBL1	**NM_182744.1**	NBL1	**NM_145888.1**	KLK10
**NM_001759.2**	CCND2	**NM_014222.2**	NDUFA8	**NM_002790.2**	PSMA5
**NM_199246.1**	CCNG1	**NM_004548.1**	NDUFB10	**NM_152255.1**	PSMA7
**NM_001239.2**	CCNH	**NM_004549.3**	NDUFC2	**NM_176783.1**	PSME1
**NM_001763.1**	CD1A	**NM_002496.1**	NDUFS8	**NM_183236.1**	RAB27A
**NM_000733.2**	CD3E	**NM_181827.1**	NF2	**NM_004583.2**	RAB5C
**NM_001769.2**	CD9	**NM_001001716.1**	NFKBIB	**NM_133629.1**	RAD51L3
**NM_005191.2**	CD80	**NM_005008.2**	NHP2L1	**NM_021033.4**	RAP2A
**NM_152942.1**	TNFRSF8	**NM_198175.1**	NM23A/NME1	**NM_002899.2**	RBP1
**NM_001244.2**	TNFSF8	**NM_000904.1**	NQO2	**NM_000976.2**	RPL12
**NM_001001392.1**	CD44	**NM_000270.1**	NP	**NM_001016.2**	RPS12
**NM_198793.1**	CD47	**NM_199185.1**	NPM1	**NM_000331.2**	SAA1
**NM_000560.2**	CD53	**NM_006172.1**	NPPA	**NM_005981.3**	TSPAN31
**NM_203330.1**	CD59	**NM_002524.2**	NRAS	**NM_002970.1**	SAT
**NM_004357.3**	CD151	**NM_004559.2**	YBX1	**NM_006745.2**	SC4MOL
**NM_001786.2**	CDC2	**NM_007105.1**	SLC22A18AS	**NM_006746.3**	SCML1
**NM_033534.1**	CDC2L2	**NM_005602.4**	CLDN11	**NM_001037.3**	SCN1B
**NM_052827.1**	CDK2	**NM_175568.1**	P2RX4	**NM_003000.1**	SDHB
**NM_000075.2**	CDK4	**NM_175081.1**	P2RX5	**NM_183352.1**	SEC13L1
**NM_078467.1**	CDKN1A	**NM_002567.2**	PBP	**NM_014563.2**	TRAPPC2
**NM_004064.2**	CDKN1B	**NM_002573.2**	PAFAH1B3	**NM_003016.2**	SFRS2
**NM_000077.3**	CDKN2A	**NM_181696.1**	PRDX1	**NM_152235.1**	SFRS8
**NM_078626.1**	CDKN2C	**NM_000281.2**	TCF1/PCBD1	**NM_004593.1**	SFRS10
**NM_005195.2**	CEBPD	**NM_032403.1**	PCDHGC3	**NM_173217.1**	ST6GAL1
**NM_001806.2**	CEBPG	**NM_002592.2**	PCNA	**NM_170679.1**	SKP1A
**NM_001809.2**	CENPA	**NM_033023.1**	PDGFA	**NM_005984.1**	SLC25A1
**NM_004365.2**	CETN3	**NM_002608.1**	PDGFB	**NM_022875.1**	SMN2
**NM_005507.2**	CFL1	**NM_213612.1**	SLC25A3	**NM_004596.3**	SNRPA
**NM_001817.1**	CEACAM4	**NM_002642.3**	PIGC	**NM_198216.1**	SNRPB
**NM_152253.1**	CHKB	**NM_002648.2**	PIM1	**NM_198220.1**	SNRPB2
**NM_013324.4**	CISH	**NM_006224.2**	PITPNA	**NM_177542.1**	SNRPD2
**NM_001281.2**	CKAP1	**NM_002653.3**	PITX1	**NM_004175.3**	SNRPD3
**NM_001284.2**	AP3S1	**NM_000929.1**	PLA2G5	**NM_022807.2**	SNRPN
**NM_001288.4**	CLIC1	**NM_001005376.1**	PLAUR	**NM_000454.4**	SOD1
**NM_001291.2**	CLK2	**NM_021910.1**	FXYD3	**NM_006943.2**	SOX12
**NM_001293.1**	CLNS1A	**NM_021105.1**	PLSCR1	**NM_001047.1**	SRD5A1
**NM_007097.2**	CLTB	**NM_153321.1**	PMP22	**NM_003132.1**	SRM
**NM_004368.2**	CNN2	**NM_174930.2**	PMS2L5	**NM_003135.1**	SRP19
**NM_007310.1**	COMT	**NM_001003686.1**	PMS2L3	**NM_003144.2**	SSR1
**NM_001300.3**	KLF6	**NM_022716.1**	PRRX1	**NM_003155.1**	STC1
**NM_001861.2**	OX4I1	**NM_002696.1**	POLR2G	**NM_177528.1**	SULT1A2
**NM_001305.3**	CLDN4	**NM_006232.2**	POLR2H	**NM_004177.3**	STX3A
**NM_001306.2**	CLDN3	**NM_006233.4**	POLR2I	**NM_004604.3**	STX4A
**NM_001307.3**	CLDN7	**NM_021129.2**	PPA1	**NM_003164.2**	STX5A
**NM_001878.2**	CRABP2	**NM_203430.1**	PPIA	**NM_177534.1**	SULT1A1
**NM_004379.2**	CREB1	**NM_000943.4**	PPIC	**NM_003166.2**	SULT1A3
**NM_001310.2**	CREBL2	**NM_177951.1**	PPM1A	**NM_181491.1**	SURF5
**NM_181571.1**	CREM	**NM_177969.1**	PPM1B	**NM_014231.3**	VAMP1
**NM_005206.3**	CRK	**NM_206877.1**	PPP1CB	**NM_014232.1**	VAMP2
**NM_005207.2**	CRKL	**NM_002710.1**	PPP1CC	**NM_005638.3**	SYBL1
**NM_001889.2**	CRYZ	**NM_006241.3**	PPP1R2	**NM_006754.2**	SYPL1
**NM_139014.1**	MAPK14	**NM_002715.1**	PPP2CA	**NM_003187.3**	TAF9
**NM_177436.1**	CSE1L	**NM_178002.1**	PR 53/PPP2R4	**NM_005643.2**	TAF11
**NM_022644.2**	CSH2	**NM_000945.3**	PPP3R1	**NM_172208.1**	TAPBP
**NM_177560.2**	CSNK2A1	**NM_005399.3**	PRKAB2	**NM_134324.1**	TARBP2
**NM_001320.5**	CSNK2B	**NM_207578.1**	PRKACB	**NM_201437.1**	TCEA1
**NM_001321.1**	CSRP2	**NM_212461.1**	PRKAG1	**NM_213648.1**	TCF7
**NM_000396.2**	CTSK	**NM_138981.1**	MAPK10	**NM_181738.1**	PRDX2
**NM_004394.1**	DAP	**NM_002756.2**	MAP2K3	**NM_201443.1**	TEAD4
**NM_020548.4**	DBI	**NM_002764.2**	PRPS1	**NM_003201.1**	TFAM
**NM_001924.2**	GADD45A	**NM_139277.1**	KLK7	**NM_174886.1**	TGIF
**NM_004083.4**	DDIT3	**NM_002774.2**	KLK6	**NM_003255.3**	TIMP2
**NM_001355.2**	DDT	**NM_213633.1**	PSG4	**NM_003270.2**	TSPAN6
**NM_030655.2**	DDX11	**NM_203287.1**	PSG11	**NM_003271.3**	TSPAN4
**NM_213566.1**	DFFA	**NM_148976.1**	PSMA1	**NM_021137.3**	TNFAIP1
**NM_000791.2**	DHFR	**NM_002789.3**	PSMA4	**NM_000363.3**	TNNI3
**NM_007326.1**	CYB5R3	**NM_002791.1**	PSMA6	**NM_005079.1**	TPD52
**NM_138281.1**	DLX4	**NM_002794.3**	PSMB2	**NM_003287.2**	TPD52L1
**NM_203316.1**	DPAGT1	**NM_002801.2**	PSMB10	**NM_199362.1**	TPD52L2

**S-Table 2.** Sequence variants

		**Homozygous alteration**				**Heterozygous alteration**			
**GenBank Accession No.**	**Gene Symbol**	**Synonymous alteration**	**NCBI SNP Database**	**Non-Synonymous alteration**	**NCBI SNP Database**	**Synonymous alteration**	**NCBI SNP Database**	**Non-Synonymous alteration**	**NCBI SNP Database**
**NM_000320**	QDPR Leu132Leu	G396A,	rs **2597775**						
**NM_000331**	SAA1			C209T, Ala70Val	**rs1136743**				
				T224C, Val75Ala	**rs1136747**				
**NM_001007**	RPS4X Leu164Leu	G492A,	**rs7580**						
**NM_001020**	RPS16 Gly5Gly	C15T,	**rs17626**						
	T27G, Ser9Ser		**rs17628**						
**NM_001047**	SRD5A1					A309AG, Pro103Pro	**rs3822430**		
						G348GA, Ala116Ala	**rs8192186**		
**NM_001320**	CSNK2B Tyr46Tyr	T138C,	**rs14365**						
**NM_001636**	SLC25A6 Phe136Phe	T408C,	**rs7205**						
**NM_001697**	ATP50 Gly36Gly	T108C,	**rs17728665**	A218AG, Lys73Arg	*				
**NM_001760**	CCND3							T775TG, Ser259Ala	**rs1051130**
**NM_001817**	CEACAM4			T668A, Val223Glu.	*				
**NM_001861**	COX4I1							G7GA, Ala3Thr	**rs17855751**
**NM_001889**	CRYZ Gly18Gly	G54A,	**rs4650284**			T138C, Gly46Gly	*		
**NM_002131**	HMGA1 Ser2Ser	T6C,	*	G49A, Glu17Lys	*				
	G78T, Arg26Arg	*		G112A, Gly38Arg	*				
	C255A, Gly85Gly	*	Pro48Leu	C143T,	*				
			C217T, Arg73Gly	*					
			C236T, A237G,	*					
			Pro79Leu G286A,	*					
			Glu96Lys						
**NM_002136**	HNRPA1 Gly248Gly	C744T,		*					
**NM_002414**	CD99					C369CT, Ala121Ala	**rs4575010**		
**NM_002642**	PIGC Gly89Gly	T267C,	**rs2230471**						
**NM_002813**	PSMD9			T50C, Val17Ala	**rs2230681**				
**NM_003144**	SSR1			C388T, His130Tyr	*				
**NM_003255**	TIMP2					G303GA, Ser101Ser	**rs2277698**		
**NM_004064**	CDKN1B							T326TG, Val109Gly	**rs2066827**
**NM_004137**	KCNMB1							G193GA, Glu65Lys	**rs11739136**
**NM_004175**	SNRPD3 Ala101Ala	T303C,	**rs3176991**						
**NM_004365**	CETN3							G28GC, Val10Leu	**rs4873**
**NM_004468**	FHL3 Pro180Pro	G540A,	**rs7366048**						
**NM_004549**	NDUFC2							C136CG, Leu46Val	**rs8875**
**NM_005171**	ATF1					C327CT, Tyr109Tyr	**rs1129406**		
**NM_005191**	CD80					G135GA,	**rs2228017**		
					Val45Val				
**NM_005301**	GPR35							G85GA, Ala29Thr. A880AC,	* rs3749172
**NM_005342**	HMGB3 Asn186Lys	C558G,	*					Ser294Arg	
**NM_005594**	NACA					T543TA, Ile181Ile	**rs4788**		
**NM_005984**	SLC25A1 Lys277Lys	A831G,	*						
**NM_006353**	HMGN4 Gly66Gly	G198A	**rs4871**						
**NM_006896**	HOXA7 Ala32Ala	T96G,	**rs2301720**	G52A, Ala18Thr	**rs2301721**				
**NM_007310**	COMT His12His	C36T,	**rs4633**	G322A, Val108Met	**rs4680**				
**NM_007311**	BZRP His53Arg			A158G, Ala68Ala	**rs6971**	G204GA,	**rs6972**		
**NM_012328**	DNAJB9 Pro61Pro	G183A,	**rs1043615**						
**NM_013332**	HIG2 Glu28Glu	A84G,	*						
**NM_014212**	HOXC11 Ser12Ser	T36G,	**rs4759315**						
**NM_014232**	VAMP2			T346A, Ser116Thr	*				
**NM_021068**	IFNA4							A146AC,	*
							His49Pro G178GC,	*	
							Gly60Arg T190TA,	*	
							Phe64Ile G187GC,		**rs3203576**
**NM_024009**	GJB3						Glu63Gln C357CT,	*	
						Asn119Asn			
**NM_033251**	RPL13			G334A, Ala112Thr	**rs9930567**				
**NM_052871**	MGC4677			G28T, Ala10Ser	**rs28673896**	C12CT, Thr4Thr	*	C109CT, Arg37Cys	*
**NM_058197**	CDKN2A	Arg54Gly		A160T,			*		
**NM_145888**	KLK10	A318C, Gly106Gly	**rs2075688**	T347C, Leu116Pro			*		
		C336G, Thr112Thr	**rs1061368**						
		G423A, Leu141Leu	**rs2075689**		`				
**NM_172200**	IL15RA			C248T, Pro83Leu	`		*		
				A337C, Thr113Pro			*		
**NM_172369**	C1QG	Gly215Glu		G644A,			*		
**NM_181571**	CREM	Ile137Thr		T410C,			*		
**NM_198970**	TEAD4			Pro194Leu				C580CT,	*
**NM_201544**	LGALS8			Met56Val.				A166AG,	**rs1041937**
				G542GC, Gly181Ala	*				

* No report found.

## Figures and Tables

**Table 1.** Sequence variants

	**Homozygous**		**Heterozygous**	
	Synonymous	Non-synonymous	Synonymous	Non-synonymous
Number of alterations	26	25	11	16

## References

[b1-bbi-2007-001] Amrani N Sachs MS Jacobson A 2006 Nat. Rev. Mol. Cell Biol 7 415 25 16723977 10.1038/nrm1942

[b2-bbi-2007-001] Boland CR Thibodeau SN Hamilton SR Sidransky D Eshleman JR Burt RW Meltzer SJ Rodriguez-Bigas MA Fodde R Ranzani GN Srivastava S 1998 Cancer Res 58 5248 57 9823339

[b3-bbi-2007-001] Calvert PM Frucht H 2002 Ann. Intern. Med 137 603 12 12353948 10.7326/0003-4819-137-7-200210010-00012

[b4-bbi-2007-001] Chamary JV Hurst LD 2005 Genome. Biol 6 R75 16168082 10.1186/gb-2005-6-9-r75 PMC1242210

[b5-bbi-2007-001] Culbertson MR Leeds PF 2003 Curr. Opin. Genet. Dev 13 207 14 12672499 10.1016/s0959-437x(03)00014-5

[b6-bbi-2007-001] Duan J Antezana MA 2003 J. Mol. Evol 57 694 701 14745538 10.1007/s00239-003-2519-1

[b7-bbi-2007-001] Eads CA Lord RV Wickramasinghe K Long TI Kurumboor SK Bernstein L Peters JH DeMeester SR DeMeester TR Skinner KA Laird PW 2001 Cancer Res 61 3410 8 11309301

[b8-bbi-2007-001] Fitch WM 1974 J. Mol. Evol 3 279 91 4416342 10.1007/BF01796043

[b9-bbi-2007-001] Giraud MF Velours J 1994 Eur. J. Biochem 222 851 9 8026496 10.1111/j.1432-1033.1994.tb18932.x

[b10-bbi-2007-001] Klambt D 1975 J. Theor. Biol 52 57 65 1152488 10.1016/0022-5193(75)90039-9

[b11-bbi-2007-001] Mori Y Yin J Rashid A Leggett BA Young J Simms L Kuehl PM Langenberg P Meltzer SJ Stine OC 2001 Cancer Res 61 6046 9 11507051

[b12-bbi-2007-001] Sachidanandam R Weissman D Schmidt SC Kakol JM Stein LD Marth G Sherry S Mullikin JC Mortimore BJ Willey DL Hunt SE Cole CG Coggill PC Rice CM Ning Z Rogers J Bentley DR Kwok PY Mardis ER Yeh RT Schultz B Cook L Davenport R Dante M Fulton L Hillier L Waterston RH McPherson JD Gilman B Schaffner S Van Etten WJ Reich D Higgins J Daly MJ Blumenstiel B Baldwin J Stange-Thomann N Zody MC Linton L Lander ES Altshuler D 2001 Nature 409 928 33 11237013 10.1038/35057149

[b13-bbi-2007-001] Samuels Y Wang Z Bardelli A Silliman N Ptak J Szabo S Yan H Gazdar A Powell SM Riggins GJ Willson JK Markowitz S Kinzler KW Vogelstein B Velculescu VE 2004 Science 304 554 15016963 10.1126/science.1096502

[b14-bbi-2007-001] Sato F Harpaz N Shibata D Xu Y Yin J Mori Y Zou TT Wang S Desai K Leytin A Selaru FM Abraham JM Meltzer SJ 2002 Cancer Res 62 1148 51 11861396

[b15-bbi-2007-001] Senior AE 1988 Physiol Rev 68 177 231 2892214 10.1152/physrev.1988.68.1.177

[b16-bbi-2007-001] Shimada Y Imamura M Wagata T Yamaguchi N Tobe T 1992 Cancer 69 277 84 1728357 10.1002/1097-0142(19920115)69:2<277::aid-cncr2820690202>3.0.co;2-c

[b17-bbi-2007-001] Sjoblom T Jones S Wood LD Parsons DW Lin J Barber TD Mandelker D Leary RJ Ptak J Silliman N Szabo S Buckhaults P Farrell C Meeh P Markowitz SD Willis J Dawson D Willson JK Gazdar AF Hartigan J Wu L Liu C Parmigiani G Park BH Bachman KE Papadopoulos N Vogelstein B Kinzler KW Velculescu VE 2006 Science 314 268 74 16959974 10.1126/science.1133427

[b18-bbi-2007-001] Wallace DC 1994 Proc. Natl. Acad. Sci. U.S.A 91 8739 46 8090716 10.1073/pnas.91.19.8739 PMC44682

[b19-bbi-2007-001] Wang Z Shen D Parsons DW Bardelli A Sager J Szabo S Ptak J Silliman N Peters BA van der Heijden MS Parmigiani G Yan H Wang TL Riggins G Powell SM Willson JK Markowitz S Kinzler KW Vogelstein B Velculescu VE 2004 Science 304 1164 6 15155950 10.1126/science.1096096

