# Supplementary figures and images for: Rarity of Somatic Mutation and Frequency of Normal Sequence Variation Detected in Sporadic Colon Adenocarcinoma Using High-Throughput cDNA Sequencing
Source: Bioinform Biol Insights. 2009 Nov 24;1:1–16. (PMC2287164)

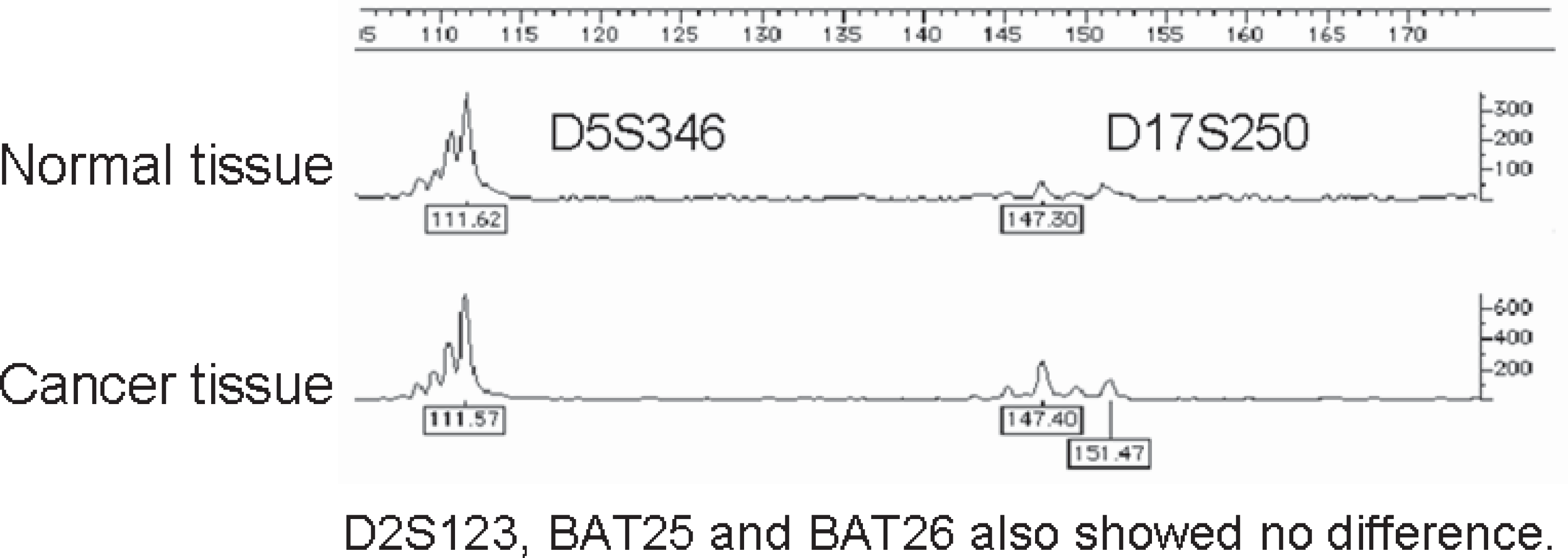

Supplement: S-Figure 1. [file bbi-2007-001f4.tif]

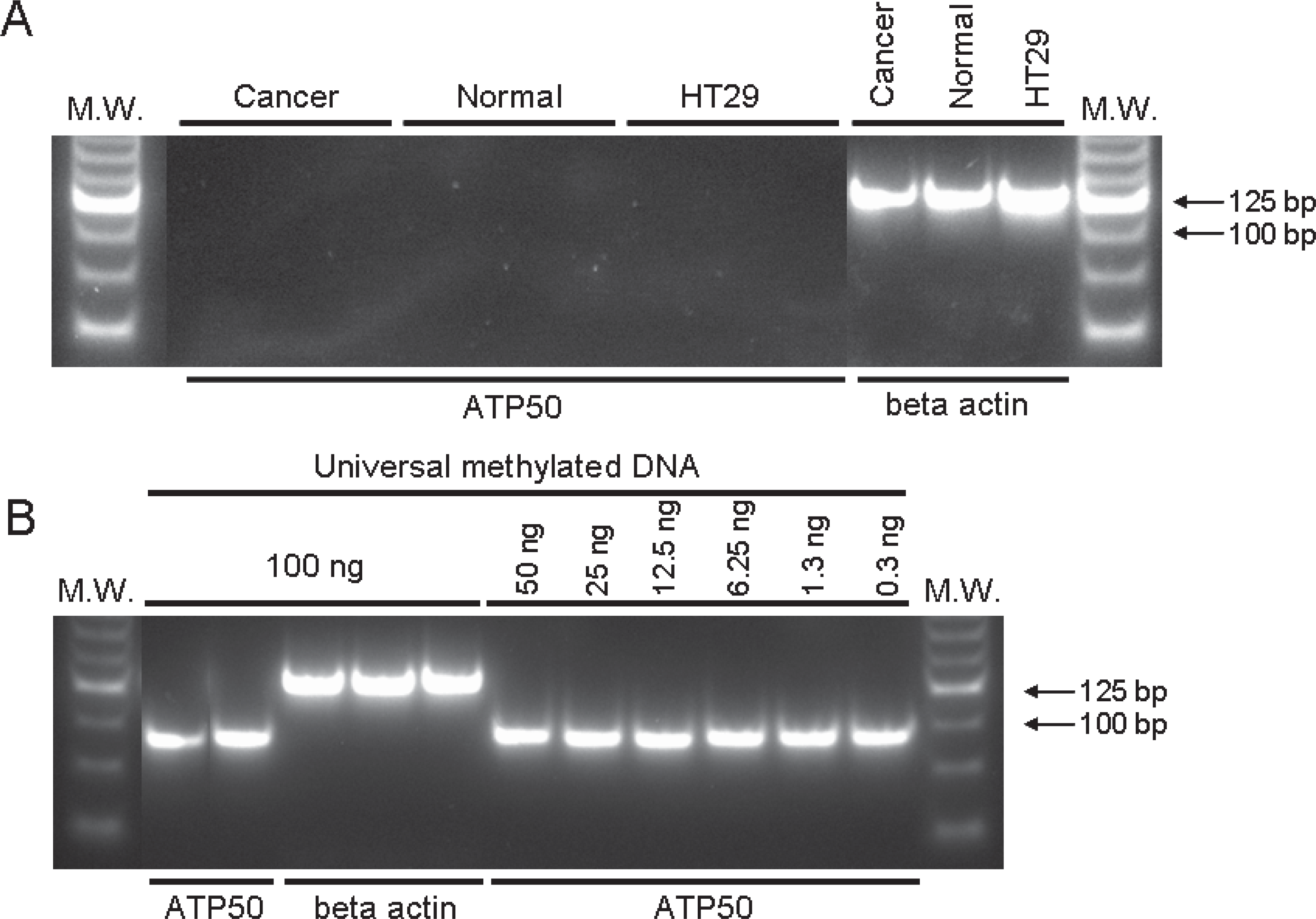

Supplement: S-Figure 2. [file bbi-2007-001f5.tif]
